# Artificial Intelligence in Nursing Support for Families: A Rapid Review

**DOI:** 10.31662/jmaj.2024-0434

**Published:** 2025-05-30

**Authors:** Michihiro Tsubaki, Kohei Kajiwara, Jun Kako, Masamitsu Kobayashi, Yoshiyasu Ito, Makoto Yamanaka, Hideaki Sakuramoto, Takahiro Kakeda

**Affiliations:** 1 School of Nursing, Kitasato University, Sagamihara, Japan; 2 Faculty of Nursing, Shimonoseki City University, Shimonoseki, Japan; 3 Graduate School of Medicine, Mie University, Tsu, Japan; 4 Faculty of Nursing, Toho University, Tokyo, Japan; 5 Faculty of Nursing Science, Tsuruga Nursing University, Tsuruga, Japan; 6 School of Nursing, Aichi Medical University, Nagakute, Japan; 7 Faculty of Nursing, Japanese Red Cross Kyushu International College of Nursing, Munakata, Japan; 8 Faculty of Nursing, Kawasaki City College of Nursing, Kawasaki, Japan

**Keywords:** artificial intelligence, family, nursing, randomized controlled trial, rapid review

Although artificial intelligence (AI) has advanced rapidly in recent years and has been applied in various fields, its use in family support has not been comprehensively reviewed. Hence, we conducted a rapid review, a form of knowledge synthesis that simplifies the systematic review process and provides timely information, to clarify the types of AI used to provide family support ^(1)^. The rapid review methodology for this study was based on the Cochrane Rapid Reviews Methods ^(2)^. This review was conducted according to the Preferred Reporting Items for Systematic Reviews and Meta-Analysis Statement ^(3)^. The protocol has been registered in the Open Science Framework (https://doi.org/10.17605/OSF.IO/6JWDZ). Studies published between January 2010 and June 2024 were included after searching PubMed, CINAHL, and the Cochrane Central Register of Controlled Trials in the Cochrane Library. The search strategy is available upon request from the corresponding author (Table 1). The search period was considering the rapid increase in research on AI technology in healthcare since 2010 ^(4)^. Furthermore, we used three search engines because rapid reviews can be conducted with a minimal number of databases. EMBASE, which specializes in medicine and pharmacy, was because this study focuses on nursing support for families. This study employed a methodology in which one reviewer initially screened the papers, and a second reviewer evaluated all excluded papers, addressing discrepancies in 20% of the cases as needed. This study took a broad view of AI interventions and included AI chatbots, predictive algorithms, and AI-equipped social robots. Primary empirical studies of any design involving AI and reporting nursing-related outcomes, as well as studies that met the following inclusion criteria, were included: (i) studies on nursing support for families; (ii) studies on any type of AI; and (iii) randomized controlled trials (RCTs), non-RCTs, prospective studies, and mixed-method studies. Studies not published in English were excluded. The quality of the included studies was appraised using the mixed-methods appraisal tool (MMAT). The MMAT is used by researchers in various disciplines to critically appraise studies with varied designs by researchers in various disciplines ^(5)^. Two independent reviewers participated in the assessment process, and the quality assessment results were reviewed by two reviewers. Discrepancies were discussed until a consensus was reached among all reviewers. Figure 1 shows the literature screening process and results. We identified 5,176 articles; however, we excluded 4,933 after reviewing their titles and abstracts. We assessed 243 full-text articles for eligibility and excluded 243 of them. We found no studies that utilized AI for family support.

**Table 1.** Search Strategy in PubMed.

Search strategy keywords	
#1	“Artificial Intelligence” [Mesh] OR “artificial intelligence” “software” [Title/Abstract] OR “machine learning software” [Title/Abstract] OR “machine learning” [Title/Abstract] OR “artificial intelligence^*^” [Title/Abstract] OR “deep learning^*^” [Title/Abstract] OR “convolutional neural network^*^” [Title/Abstract] OR “algorithm^*^” [Title/Abstract] OR “artificial neural network^*^” [Title/Abstract] OR “machine learning^*^” [Title/Abstract] OR “heuristic^*^” [Title/Abstract] OR “automated reasoning^*^” [Title/Abstract] OR “machine reasoning^*^” [Title/Abstract] OR “computer reasoning^*^” [Title/Abstract] OR “computer intelligence^*^” [Title/Abstract] OR “automated intelligence^*^” [Title/Abstract] OR “machine intelligence^*^” [Title/Abstract] OR “automated pattern recognition^*^” [Title/Abstract] OR “feature detection^*^” [Title/Abstract] OR “cade” [Title/Abstract] OR “cadx” [Title/Abstract] OR “computer aided detect^*^” [Title/Abstract] OR “computer aided diagnos^*^” [Title/Abstract] OR “computer aided classif^*^” [Title/Abstract]
#2	“nurs^*^” [Title/Abstract]) OR “Nursing” [Mesh]
#3	#1 AND #2
#4	Filters applied: from 2010_-_2024
#5	#4 AND English
#6	#5 AND Humans

**Figure 1.**
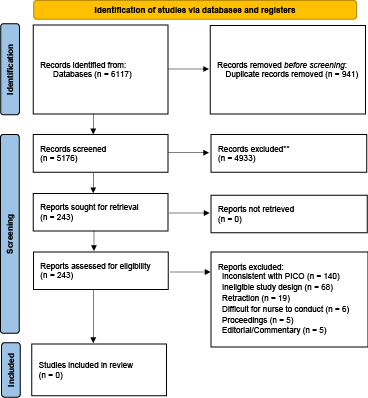
Flowchart of the study selection process.

AI has been highlighted in almost all scientific fields. It is expected that this technology will also be applicable in the field of family support. To our knowledge, this is the first study to clarify the application of AI in the field of family support. The results of this study showed that despite advances in AI, there have been no intervention studies using AI aimed at supporting families. One reason may be that family support is primarily psychological and social, relying heavily on emotions and relationships, which may be difficult to perfectly reproduce using AI. On the other hand, studies on patients are conducting RCTs in various fields to evaluate AI interventions. The characteristics of AI intervention research targeting patients varied depending on the study period. Since around 2015, clinical research using the mental commitment robot “PARO” began to appear ^(6), (7), (8)^. This robot can learn subjects’ movements and environment and respond according to the situation. All these studies were RCTs targeting older adults with dementia, and it was reported that the robot reduced patients’ agitation and depression and eventually the amount of psychotropic medication needed. In 2023, research involving the use of chatbots emerged ^(9), (10)^. Chatbots have emerged as promising platforms for delivering education to help patients manage their symptoms. These aforementioned studies used chatbots for interventions pertaining to self-care and smoking cessation for patients with breast cancer. In recent years, ChatGPT has been used in research. Preoperative ChatGPT-based interventions have been reported to reduce patient anxiety ^(11)^. AI applications in the medical field began with its use to provide commercial services to the general population, and recently, it has moved toward proposing highly personalized solutions for individuals through interactive methods such as chatbots. In 2025, the effectiveness of a generative AI chatbot targeting dementia caregiving among family caregivers has been reported, and this study took a broad view ^(12)^. As such, research using AI is developing rapidly; however, the subjects of such studies are always patients. As medical care becomes more advanced, there are an increasing number of situations in which family support is important. However, AI, a popular technology in recent years, has not yet penetrated the family support field. Its application in the field is expected to contribute to the further development of medical care. Furthermore, rapid reviews, which allow for a timely understanding of current trends, are effective in such fields ^(13)^. In conclusion, there is limited evidence on the use of AI in the field of family support. The application of AI in patient care is evolving, and we hope to foster its use in supporting families and patient-targeted interventions. This study is a rapid review and is appropriate for a new field such as this one. In this study, we purposely set a search scope that would allow us to understand the current situation. Hence, the fact that no research was found to be applicable is a limitation of this study.

## Article Information

### Conflicts of Interest

None

### Sources of Funding

This study was supported by JSPS KAKENHI (grant number: JP21K17428).

### Acknowledgement

We thank Editage (www.editage.jp) for English-language editing.

### Author Contributions

Kohei Kajiwara and Jun Kako designed this study. Michihiro Tsubaki, Kohei Kajiwara, Jun Kako, Masamitsu Kobayashi, Yoshiyasu Ito, Makoto Yamanaka, Hideaki Sakuramoto, and Takahiro Kakeda conducted screening of the title, abstract, and the full text screening of the included studies. Michihiro Tsubaki drafted the initial manuscript, after which discussions were conducted with other authors (Kohei Kajiwara, Jun Kako, Masamitsu Kobayashi, Yoshiyasu Ito, Makoto Yamanaka, Hideaki Sakuramoto, and Takahiro Kakeda). All authors reviewed and approved the final manuscript.

### Approval by Institutional Review Board (IRB)

Since the data used in this study were extracted from previously published articles, ethical approval and informed consent were deemed unnecessary.
